# Outcomes of Simulation in Resident Imaging-Guided Breast Biopsy Training

**DOI:** 10.7759/cureus.16815

**Published:** 2021-08-01

**Authors:** Shahad Al Bayati, Martin A Dufwenberg, Colin O'Brien, Brian D Skidmore, Kimberly A Fitzpatrick, Marisa H Borders

**Affiliations:** 1 Department of Medical Imaging, The University of Arizona College of Medicine - Tucson, Tucson, USA; 2 Department of Family Medicine, PeaceHealth Southwest Medical Center, Vancouver, USA; 3 Department of Radiology, University of Colorado School of Medicine, Aurora, USA; 4 Breast Imaging, Radiology Ltd., Tucson, USA

**Keywords:** simulation in medical education, radiology teaching, breast biopsy, medical resident education, breast lesions and imaging modality

## Abstract

Introduction

We evaluate diagnostic radiology residents’ perceptions of an ultrasound-guided and stereotactic breast biopsy simulator used at an academic medical center. This simulator is low-cost and easily reproducible. We aim to understand if this simulator improves residents’ self-reported confidence in performing breast biopsy procedures on live patients.

Methods

Twenty-eight diagnostic radiology residents were instructed in how to perform ultrasound-guided breast biopsies and stereotactic breast biopsies using real biopsy and imaging equipment, but with tissue models in lieu of live persons. The hands-on experience was preceded by a didactic lecture. The ultrasound-guided tissue model was created with blueberries that were inserted in tofu, and the stereotactic tissue model was created by placing crushed calcium carbonate tablets into cored eggplant. Residents were asked to fill out a survey before and after participating in the simulation, where they self-reported their confidence level at performing ultrasound-guided and stereotactic breast biopsies.

Results

Twenty-eight diagnostic radiology residents participated in the simulation. All residents completed the pre-simulation survey and of these residents, twenty-one completed the post-simulation survey. Prior to the simulation residents reported a median confidence level of 3.5 out of 10 in performing ultrasound-guided breast biopsies, and a median confidence level of 1.0 out of 10 in performing stereotactic-guided breast biopsies. After the simulation, residents reported a median confidence level of 7.0 out of 10 in performing ultrasound-guided breast biopsies, and a median confidence level of 3.0 out of 10 in performing stereotactic-guided breast biopsies. Increases in resident confidence level were statistically significant for both biopsy types (p < 0.01).

Conclusion

Simulated biopsies can increase the confidence of diagnostic radiology residents that are learning to perform breast biopsies before they perform real biopsies on live patients. Providing simulation training and thereby improving resident confidence may help reduce physician error and patient harm due to poor biopsy techniques.

## Introduction

Percutaneous core needle biopsy as a next step following concerning imaging findings has been recognized as superior to surgical biopsy as it is minimally invasive, faster, and less expensive [1]. As radiologists perform both ultrasound-guided and stereotactic biopsies, it is important that diagnostic radiology (DR) programs provide adequate training opportunities in both modalities for their residents. 

Unfortunately, without hands-on training, novice residents may feel that they lack the confidence to perform their first biopsy on a patient. Simulation has become one of the most important teaching tools in medical education as it allows learners to apply theoretical knowledge in a safe and realistic setting [2]. Multiple medical disciplines now use hands-on simulations in their curricula and previous studies have shown that these simulations improve student confidence, understanding, and satisfaction [3].

Simulation in DR education has a rich history, most notably with the classic case conference where a resident walks an attending physician through their thought process as they review imaging from a previous case [4]. More recently DR programs have begun to use hands-on simulation for learning procedures. For example, Mandiratta-Lala, et al. used a simulation-based training curriculum to teach ultrasound-guided biopsies and concluded that controlled simulation-based training can be an invaluable tool to improve the knowledge level, dexterity, and confidence of residents performing ultrasound-guided procedures [5].

The purpose of this study is to evaluate the effectiveness of simulation in improving DR resident confidence level in performing ultrasound-guided and stereotactic breast biopsies. Our approach is in a similar vein to that of Mandiratta-Lala et al. but differs in that our models are created from common household items. This approach is advantageous because it makes the simulations easy to implement in any DR training program, as long as the program has access to the biopsy equipment needed to perform a live image-guided biopsy.

## Materials and methods

Twenty-eight DR residents at an academic medical center participated in a simulated ultrasound-guided breast biopsy and a simulated stereotactic breast biopsy training program. This consisted of a didactic lecture followed by a hands-on training session for each biopsy technique. 

The residents were asked to fill out an anonymous survey before and after the exercise, which asked them to evaluate their confidence with both procedures on a 10 point Likert scale. Residents were also asked about their prior experiences with breast biopsies and what year of the residency program that they were in. 

Descriptive statistics were calculated for pre- and post-simulation surveys. Because surveys were anonymous, we could not match pre- and post-simulation surveys for individuals. As such we could not use a paired sample statistical analysis method. Therefore, pre- and post-simulation surveys were compared using the Mann-Whitney U Test at a significance level of 0.01.

Ultrasound-Guided Biopsy Tissue Model

**Figure 1:**
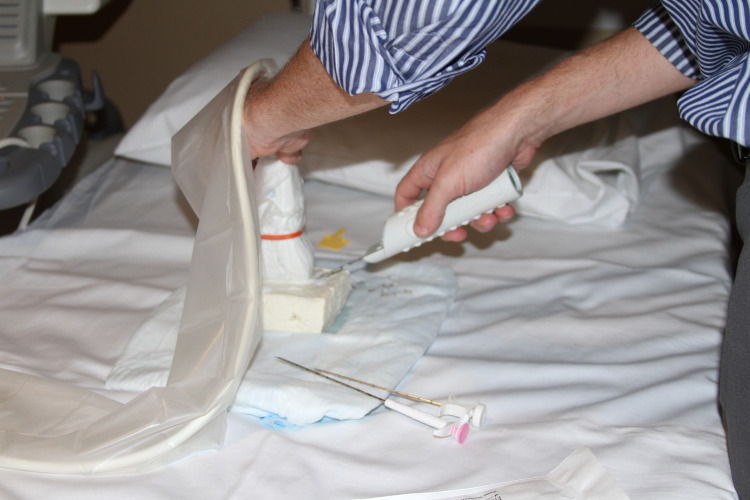
A resident using the tofu and blueberry model to practice ultrasound-guided breast biopsies.

**Figure 2:**
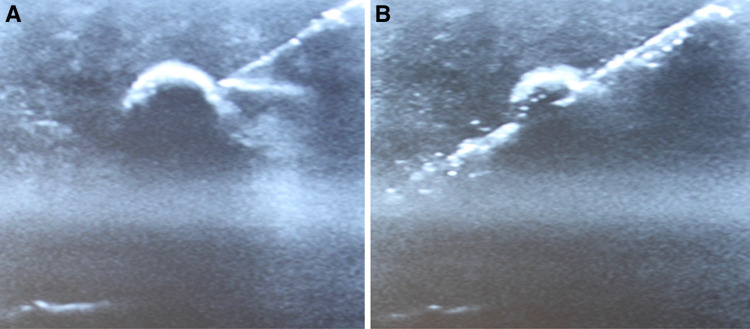
Pictures from the ultrasound-guided biopsy simulation. Figure 2A shows a biopsy needle in the tofu phantom adjacent to a blueberry, which represents a breast mass. Figure 2B shows the needle entering the blueberry, simulating an ultrasound-guided breast biopsy.

A block of firm or extra-firm tofu was used to simulate breast tissue and small hard blueberries were used to simulate tumors to be biopsied. Tofu blocks were wrapped in paper towels, placed in a glass container, and stored in a refrigerator for up to 3 weeks prior to the simulation training. Small ¼ inch incisions were made in the side of the tofu block and 4-6 blueberries were gently inserted. Figure 1 shows a resident practicing ultrasound-guided breast biopsies on the tofu and blueberry tissue model. Figure 2 shows the sonographic appearance of the phantom model during the simulation.

Stereotactic Biopsy Tissue Model

**Figure 3:**
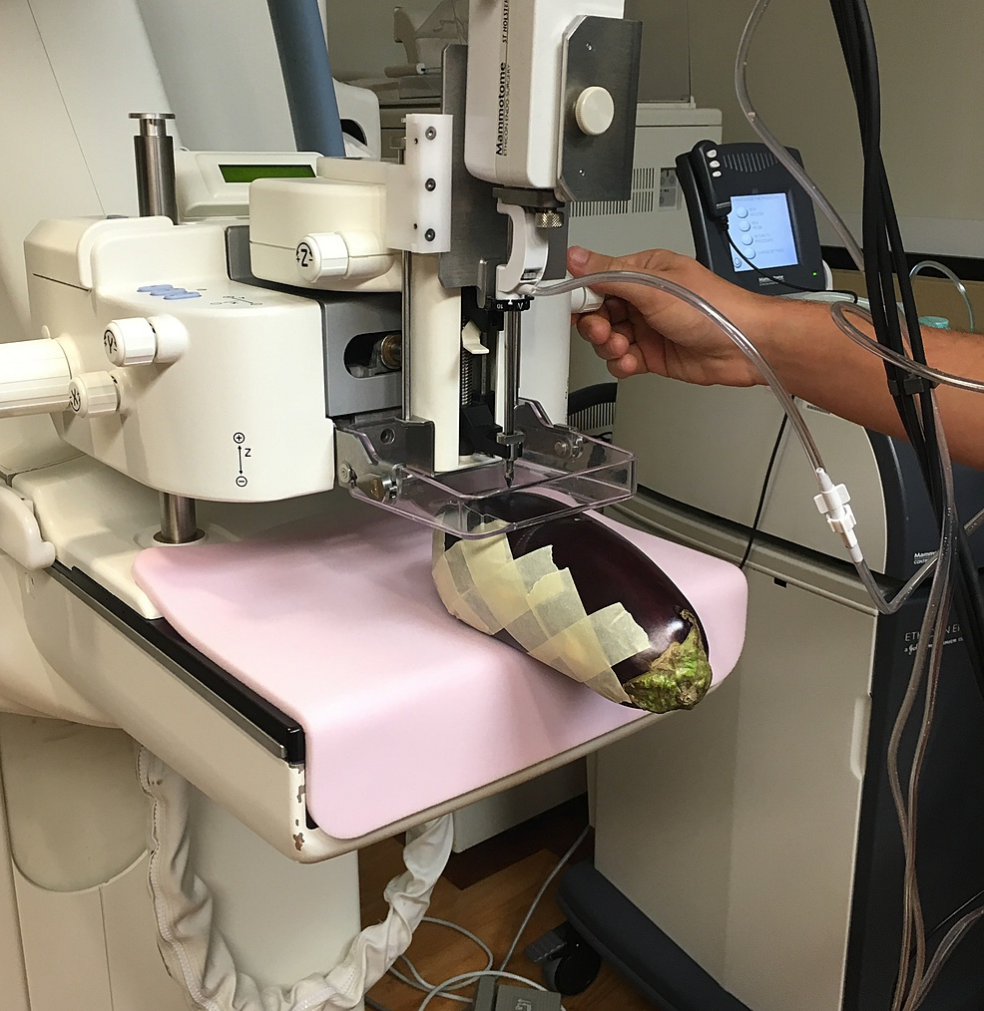
A resident practicing stereotactic breast biopsy technique using the eggplant tissue model.

**Figure 4:**
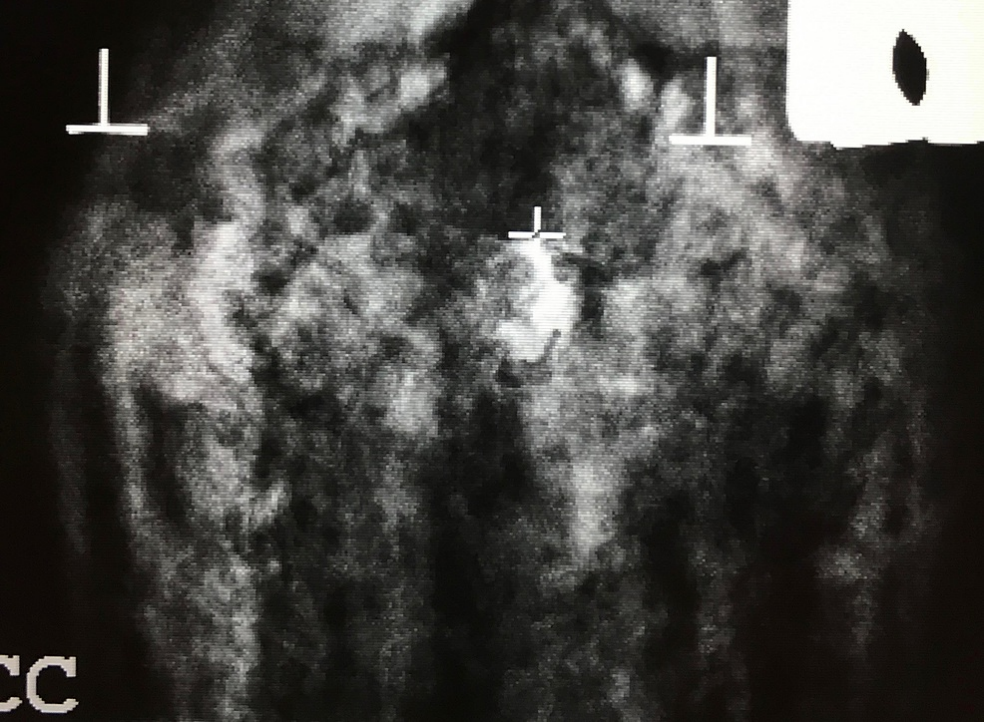
Radiographic picture of the eggplant phantom taken during the stereotactic breast biopsy simulation. Note the target in the center that demarcates the location of the crushed calcium carbonate tablets, which represent breast calcifications in the simulation.

Using fresh eggplant, phantoms were made by removing a small core with a straw and inserting a small amount of crushed calcium carbonate tablets to simulate microcalcifications. The eggplant core was then replaced to contain the calcium carbonate pieces. The defect on the "skin" was then covered with a piece of tape to create a seal so the vacuum suction would function properly. The eggplant was then positioned in compression, just like a breast, and targeted for biopsy using imaging to identify the “calcifications” prior to performing the biopsy. Overall, a single medium-sized eggplant makes 3-4 practice targets. Figure 3 shows a resident using the eggplant tissue model to practice stereotactic breast biopsy. Figure 4 shows the radiographic appearance of the eggplant phantom during the simulation.

## Results

A total of twenty-eight DR residents participated in the study. Of those, seven (25.0%) were in their first year of residency, five (17.9%) were in their second year of residency, seven (25.0%) were in their third year of residency, and nine (32.1%) were in their fourth year of residency. Eleven (39.3%) participants had never completed a breast imaging rotation in the past, while seventeen (60.7%) had completed at least one breast imaging rotation prior to this study. All twenty-eight residents completed the pre-simulation survey which asked them to rate their confidence in performing ultrasound-guided and stereotactic biopsies on a Likert scale from 1 to 10. Twenty-one residents completed the post-simulation survey.

Pre-simulation, residents reported a median confidence level of 3.5 out of 10 (mean 4.6 +/- 0.6) in performing ultrasound-guided breast biopsies. Post-simulation, residents reported a median confidence level of 7.0 out of 10 (mean 7.0 +/- 0.5) in performing ultrasound-guided breast biopsies. This represented a statistically significant (Mann-Whitney U = 157, n1 = 28, n2 = 21, p = 0.006<0.01, two-tailed; Table 1) change in resident confidence.

**Table 1:** Descriptive statistics of resident confidence before and after ultrasound-guided breast biopsy simulation.

	Pre-simulation	Post-simulation
Median reported confidence	3.5	7.0
Mean reported confidence	4.6	7.0
Standard deviation of the mean	0.6	0.5
95% confidence interval	1.1	1.0
Variance	8.5	4.6
Responses	28	21

Pre-simulation, residents reported a median confidence level of 1.0 out of 10 (mean 2.0 +/- 0.3) in performing stereotactic-guided breast biopsies. Post-simulation, residents reported a median confidence level of 3.0 out of 10 (mean 3.7 +/- 0.5) in performing stereotactic-guided breast biopsies. This also represented a statistically significant (Mann-Whitney U = 147, n1 = 28, n2 = 21, p = 0.003<0.01, two-tailed; Table 2) change in resident confidence. 

**Table 2:** Descriptive statistics of resident confidence before and after stereotactic-guided breast biopsy simulation.

	Pre-simulation	Post-simulation
Median reported confidence	1.0	3.0
Mean reported confidence	2.0	3.7
Standard deviation of the mean	0.3	0.5
95% confidence interval	0.5	1.0
Variance	2.0	4.7
Responses	28	21

Inferential statistics for the two breast biopsy simulations are summarized in Table 3. 

**Table 3:** Inferential statistics of resident confidence before and after breast biopsy simulations.

	Ultrasound-guided biopsy	Stereotactic biopsy
Mann Whitney test statistic	157	147
Z-statistic	-2.8	-3.0
p-value	0.006	0.003

## Discussion

The data collected suggests that providing simulation training in ultrasound-guided and stereotactic breast biopsies for DR residents positively influences the residents’ confidence and perceived ability in performing these procedures. There was a statistically significant increase in overall resident confidence level in performing biopsies using each modality after they practiced on the simulated tissue. Based on these data, it is likely that a major source of resident anxiety and low confidence in performing ultrasound-guided and stereotactic breast biopsy procedures is inexperience and minimal hands-on practice using these techniques.

The goal of this simulation training was to increase resident confidence in performing ultrasound-guided and stereotactic breast biopsies, which may translate to the clinical setting and improve patient care. Therefore, the results of this study suggest that offering procedure-related training in a simulated environment may benefit residents in DR training programs.

Although there was a statistically significant improvement relative to baseline, after the stereotactic biopsy simulation DR residents reported a low confidence level of three out of ten. To improve the stereotactic biopsy simulation we suggest that residents also determine the precise x-, y-, and z- coordinates for the biopsy location. In our stereotactic biopsy simulation, the coordinates for the calcifications in the phantom had already been localized by a technician, and the residents only had to advance the needle to the appropriate biopsy location. By having residents localize the coordinates for the biopsy site without assistance, they would gain a more sophisticated understanding of the equipment and procedure and thereby become more confident with stereotactic biopsies.

An advantage of our simulation approach is that it gives residents an opportunity to familiarize themselves with standard biopsy equipment like needles and ultrasound. Another advantage is that our approach is simple and affordable to implement in any DR program that has access to ultrasound and stereotactic biopsy equipment. A disadvantage of our simulation is that it does not involve real patients. With real patients, radiologists must provide empathy and comfort, navigate language barriers, and be mindful of small talk between them and their staff. We suggest that to improve the simulation real patients that have undergone breast biopsy could be invited to the didactic portion of the simulation to briefly talk about their experience. This could help residents understand the emotional needs of their patients while undergoing a biopsy.

Future studies should work to collect objective data instead of only subjective data. For example, instead of asking residents about their confidence level with the biopsy procedures, an evaluator could assess the residents on their technical ability in performing the procedures. We also suggest that future simulation programs be implemented in such a way that residents undergo simulation training just prior to their breast imaging rotation, instead of as a once per year event. This would ensure that practice and application of breast biopsies go hand-in-hand.

There are limitations to this study. First, the data collected measure only the participants’ self-reported confidence rather than an objective evaluation of correct biopsy technique by a qualified examiner. Therefore, we cannot draw conclusions regarding the effect of simulation training on resident skill in performing these biopsy techniques. Second, we did not stratify the pre-simulation survey responses and post-simulation survey responses based on resident training level. It is possible that the simulation training had a smaller impact on participants with a higher level of training and who had completed one or more breast imaging rotations. Third, we received twenty-eight pre-simulation survey responses and only twenty-one post-simulation survey responses. Thus, the data may be impacted by attrition bias. Fourth, because surveys were anonymous, we could not match up individual pre- and post-simulation surveys. As such, we could not use a paired sample statistical analysis method and had to assume independent sampling for our inferential statistics.

## Conclusions

Simulation has become a mainstay of medical education and research across medical specialties has shown that medical simulation improves student confidence and understanding. Our results for breast biopsy simulation among DR residents using inexpensive and easily reproducible models to simulate ultrasound-guided and stereotactic biopsies are consistent with these prior findings. After receiving hands-on training using tofu and eggplant simulation phantoms, DR residents reported a statistically significant increase in self-reported confidence using both modalities, thereby providing more support for the utility of simulation in medical training.
